# Changes in Mental Health during the COVID-19 Pandemic among Representative Sample of Young Adults from Germany, Israel, Poland, and Slovenia: A Longitudinal Study

**DOI:** 10.3390/ijerph19105794

**Published:** 2022-05-10

**Authors:** Dominika Ochnik, Ana Arzenšek, Aleksandra M. Rogowska, Urša Mars Bitenc, Joy Benatov

**Affiliations:** 1 Faculty of Medicine, University of Technology, 40-555 Katowice, Poland; 2 Faculty of Management, University of Primorska, 6101 Koper, Slovenia; ana.arzensek@fm-kp.si; 3 Institute of Psychology, University of Opole, 45-052 Opole, Poland; arogowska@uni.opole.pl; 4 Department of Psychology, Faculty of Mathematics, Natural Sciences and Information Technologies, University of Primorska, 6101 Koper, Slovenia; ursa.mars@upr.si; 5 Department of Special Education, University of Haifa, Haifa 3498838, Israel; jbentov2@gmail.com

**Keywords:** COVID-19, young adults, mental health, longitudinal, cross-national

## Abstract

The aim of this cross-national longitudinal study was to identify a change in mental health indicators: coronavirus-related post-traumatic stress disorder (PTSD), perceived stress, and fear of vaccination (FoVac). The first measurement (T1) took place in February 2021, and the second (T2) took place in May–June 2021. The sample consisted of 1723 participants across Germany, Israel, Poland, and Slovenia, between the age of 20 and 40 (*M* = 30.74, *SD* = 5.74). A paired-samples Student’s *t*-test was used for testing the differences between T1 and T2. A repeated measures two-way ANOVA was performed to examine changes over time (T) and across the countries (C). A significant although small decrease at T2 was found for coronavirus-related PTSD, perceived stress, and FoVac. A significant main effect was found for T, C, and TxC for all variables, except the interaction effect for coronavirus-related PTSD and perceived stress. A medium effect size was found for coronavirus-related PTSD and FoVac across countries as well as perceived stress over time. A small effect size was revealed for coronavirus-related PTSD and FoVac over time, perceived stress across countries, and interaction for FoVac. A significant improvement in mental health was demonstrated across the four countries (particularly in Israel); however, there were still differences among each of them. Therefore, the cross-national context should be taken into consideration when analyzing the effects of the COVID-19 pandemic on mental health.

## 1. Introduction

Coronavirus disease 2019 (COVID-19) has had a detrimental effect on people’s health all over the world. An atmosphere of negative emotions was generated globally, due to disrupted life rhythms, including stay at home orders; social distancing; the closing of schools, shops, and entertainment venues; working from home; limited access to sport and cultural events; cancellation of travel; and media overload [1]. While all of these public health interventions may benefit the general public, they can become a psychological challenge for those who are isolated, their loved ones, and health care personnel [2]. Various distress reactions may follow, such as anxiety, depression, insomnia, diminished perception of safety, and anger, as well as increased health risk behaviors, such as alcohol or drug abuse. Additionally, measures to combat the virus may cause or deepen existing family or social conflicts, altered work–life balance, and loneliness. 

Many countries have begun to monitor the pandemic’s effect on mental health. Badellino et al. [3] found signs of psychological distress in 62.4% of all respondents after only nine days, following the mandatory quarantine in Argentina. An early study on COVID-19 mental health consequences by Bäuerle et al. [4] noted a rise in distress (65.2%), COVID-19-related fear (59%), generalized anxiety (44.9%), and depression (14.3%) among German residents. An in-depth study by Qiu et al. [5] noted that around 35% of people were psychologically affected by the pandemic. Czeisler et al. [6], further, observed a higher increase in insomnia, anxiety disorders, and dementia among patients who were hospitalized for COVID-19, compared to those who were admitted to the hospital for other reasons. 

Recent surveys reported that greater mental health risk was linked to the female gender, young age, low educational level, being without a job due to the pandemic, and physical and psychological comorbidities [7,8,9]. Cross-sectional research on mental health among young adults from nine countries during the first wave of the COVID-19 pandemic showed that female gender and physical activity were not universal predictors of anxiety and depression, and that these predictors depended on the cross-cultural context [10]. 

The cross-cultural context seems to be particularly important for understanding challenges associated with coronavirus-related PTSD. Research among 1741 students from Germany, Poland, Russia, Slovenia, Turkey, and Ukraine clearly showed that exposure to COVID-19 significantly differed between countries as well as the two first waves of the pandemic [11]. The prevalence of coronavirus-related PTSD risk at two cutoff scores (44 and 50) was 32.70% and 23.10%, respectively, in the total sample. High and very high coronavirus-related PTSD risk was associated with some aspects of exposure to the COVID-19 pandemic, i.e., a loss of friends/relatives, job loss, and worsening economic status due to COVID-19. Therefore, direct exposure to COVID-19, such as experiencing symptoms, testing, or being hospitalized for coronavirus, was not a risk factor for coronavirus-related PTSD. However, indirect exposure to the COVID-19 pandemic—restrictions and consequences of those restrictions—was related to coronavirus-related PTSD. Hence, experiencing exposure to the COVID-19 pandemic, and not to the disease itself, significantly predicted coronavirus-related PTSD in this repeated cross-sectional study among students from six countries. Considering that those aspects of exposure to the pandemic differed significantly among countries, the cross-cultural context is vital for explaining changes in mental health during the pandemic. 

Research from the USA showed that young adults were among the groups most at risk during the COVID-19 pandemic [6,12]. These findings are consistent with research from non-US samples. For example, Pant Bisht et al. [13] found that lockdown-related stress varies by age group, with older citizens in India (50+) experiencing less stress than younger people (<50). Likewise, Huang and Zhao [14] found in their study that people under the age of 35 have a higher incidence of depressive symptoms and anxiety than people over the age of 35. Younger individuals who spent a lot of time worrying about the outbreak were more likely to manifest psychological effects. Likewise, a study that compared quality of life, well-being, and life satisfaction among Polish and German older adults during the COVID-19 pandemic found that quality of life, well-being, and life satisfaction were rated more favorably among older participants. Compared to young people, older people also reported lower trait anxiety levels and coronavirus threat, as well as greater optimism, sleep quality, and risk tolerance; they also found relaxation easier than middle-aged respondents [15]. A study carried out in Israel by Levkovich [1] shows the COVID-19 pandemic had different impacts on people of different ages. In the first stage of the pandemic, negative emotional responses were substantially higher among participants under the age of 30 than among adult participants of all other age groups. Similar results in a sample from Israel were observed in a study by Horesh et al. [16].

As a result, there is a pressing need to learn more about the psychological effects of the COVID-19 pandemic, particularly among young adults.

Due to the rapid spread of COVID-19, having a high rate of vaccination in a population was crucial to at least slow down this spread. Therefore, it was vital to understand attitudes towards COVID-19 vaccination. The concept of vaccination hesitancy has been widely recognized and measured through various methods [17,18,19]. However, a rarely explored issue is fear of vaccination [20]. Recent research has shown that it is more strongly related to intention to vaccinate than fear of COVID-19 [20]. However, little is known regarding the concept of fear of vaccination. Therefore, our study aims to fill this gap. 

In previous research, we have revealed the prevalence of coronavirus-related PTSD and perceived stress among representative samples of young adults from Germany, Israel, Poland, and Slovenia [21]. The variables were dichotomized to show the percentage of the population with and without risk of coronavirus-related PTSD and perceived stress. We showed that the highest prevalence of coronavirus-related PTSD was in Germany, while the lowest was in Israel. In a comparison of different countries, the highest rate of perceived stress at two measurement timepoints was revealed in a Polish sample. A higher risk of coronavirus-related PTSD was associated with younger age (20–30 vs. 31–40), being single, and having children. The risk of high perceived stress was associated with the female gender, being single, and having children. 

The aim of this study is to show changes in mental health indices over a three-month period during the third wave of the COVID-19 pandemic, such as coronavirus-related PTSD and perceived stress, and changes in key variables related to the COVID-19 pandemic, such as fear of vaccination. Therefore, in this paper, all variables are continuous. Furthermore, we aim to reveal the effects of the country (Germany, Israel, Poland, Slovenia), time of measurement (two measurements in a three-month period) and interaction between country and measurement time for mental health indices (coronavirus-related PTSD, perceived stress, fear of vaccination) among representative samples of young adults that are particularly susceptible to mental health deterioration during the pandemic. The countries in our study represent cultural diversity in terms of traditional vs. secular and survival vs. self-expression values. The Inglehart–Welzel World Cultural Map [22] gathers all countries and categorizes them into eight clusters based on the dimensions of certain values. Three out of eight value clusters are represented in our study. Catholic Europe is represented by Poland and Slovenia, Protestant Europe by Germany, and West and South Asia by Israel; these countries represent a range of diverse global cultural values. 

While most studies have used a cross-sectional approach, the present study is among only a few to implement a longitudinal and cross-cultural methodological design based on representative national samples. This new knowledge will allow us to recommend tailored approaches, reducing the pandemic’s potential mid- and long-term psychological negative consequences in these selected countries.

## 2. Materials and Methods

### 2.1. Study Design

The data were collected by the ARIADNA panel, gathering representative samples of young adults between 20 and 40 years old in Germany, Israel, Poland, and Slovenia through a longitudinal cross-national study design. The samples were representative of gender, student status, and employment status in each country to address potential sources of bias. The participants were enrolled into a rewards system (points exchanged for prizes, cash, or charity donations).

The survey was conducted online and prepared in the native language of each country. Translators and experts from each country translated the survey questions from English, in line with cross-cultural adaptation standards [23,24]. The participants answered all questions, as filling in each response was required to continue the survey. The participants could stop at any moment and return to finish the survey at a later time, and there was no time limit. The average duration of data collection was 21.52 min (*SD* = 136.75). 

There were 2951 participants at the first measurement point of the study (T1). However, at the second measurement (T2) point, 1724 participants responded out of the 2951 in T1. The response rate was 58.42% in T2. After performing anomaly detection, one observation was excluded from T2. Hence, the final total sample consisted of 1723 participants from Germany, Israel, Poland, and Slovenia.

#### The COVID-19 Pandemic Situation in Poland, Slovenia, Israel, and Germany during the Study

This longitudinal study was conducted in two waves over a three-month period. The first measurement of the study (T1) took place between 19 and 26 February 2021, whereas the second measurement (T2) was between 26 May and 9 June 2021.

The data were collected between the second and third waves of the COVID-19 pandemic in Poland and Germany, with the third wave starting in March 2021. In Israel, the third pandemic wave finished in April 2021, while the fourth began in June 2021. Slovenians experienced a national lockdown from 1 to 11 April 2021. Therefore, although the period between the study’s measurement points was relatively short (three months), the pandemic situation drastically changed in each country during this period.

**Figure 1:**
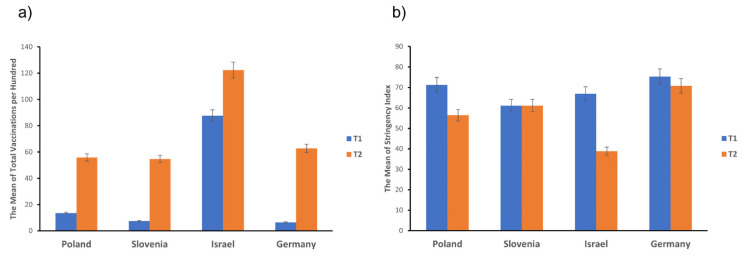
The mean scores at the first measurement (T1) in February 2021 and the second measurement (T2) in May–June 2021 of: (**a**) total vaccinations per hundred in Poland, Slovenia, Israel, and Germany; calculation based on Our World in Data panel [25]. (**b**) The Stringency Index; calculation based on the Oxford COVID-19 Government Response Tracker (OxCGRT) [25,26].

Another vital aspect of the pandemic situation during the study is the number of vaccinated citizens in Poland, Slovenia, Israel, and Germany. The mean of total vaccinations per hundred people [25] during T1 and T2 showed that the number of total vaccinations visibly increased in each country. However, the data for Israel exceed the number of vaccinated citizens, especially during T1. Details are presented in Figure 1a. We also analyzed the strictness of government restrictions with the mean scores of the Stringency Index (SI) during T1 and T2 [25,26]. SI measures the stringency of restriction during the COVID-19 pandemic (ranging from 0 to 100) and consists of nine indicators: restrictions on public gatherings, closures of public transport, stay-at-home requirements, the cancellation of public events, public information campaigns, internal traffic restrictions, and international travel control. The analyses showed that in Slovenia, the mean SI value was stable during T1 and T2, while it declined during T2 in Poland, Israel, and Germany, as presented in Figure 1b. SI is related to mental health, particularly to a higher risk of depression [27].

Data regarding daily new confirmed COVID-19 deaths per million people [25] showed that there was a negative relative change between T1 and T2 in all countries, ranging from −55% in Poland, to −81% in Germany, to −86% in Slovenia, and to −100% in Israel.

### 2.2. Participants

A representative sample of 1723 adults from Poland (*n* = 446, 26%), Slovenia (*n* = 431, 25%), Israel (*n* = 428, 25%), and Germany (*n* = 418, 24%) participated in the study. The mean age of participants was 31 (ranging in age between 20 and 40 years, *M* = 30.74, *SD* = 5.74). Among adults, 54% were women (*n* = 935), 58% were childfree (*n* = 1001), and 75% (*n* = 1297) lived in towns or cities (vs. villages). The majority of respondents (71%, *n* = 1218) indicated their work status as employed (77%, *n* = 1324) and relationship status as in a couple (vs. single, 29%, *n* = 505). The current student’s status represented 24% of the total sample (*n* = 420).

### 2.3. Measurements

#### 2.3.1. Coronavirus-Related PTSD

The 17-item PTSD Checklist-Specific Version (PCL-S) [28] was used to evaluate coronavirus-related PTSD. It is a five-point Likert scale ranging from 1 to 5 (1 = not at all; 2 = a little bit; 3 = moderately; 4 = quite a bit; 5 = extremely). The total score ranges from 17 to 85. Scores from 17 to 29 are interpreted as no severity; 28–29 as some PTSD symptoms; 30–44 as moderate to highly moderate severity; and 45–85 as high severity of PTSD symptoms. PCL-S gives us the opportunity to evaluate the risk of PTSD related to a specific stressful event, which, in this case, is the COVID-19 pandemic. Therefore, we have used this version of the scale, even though it is based on the Diagnostic and Statistical Manual of Mental Disorders, fourth edition (DSM-4) [29]. Furthermore, the latest version, PLC-5 [30], based on DSM-5 [31], is very similar to PCL-S; however, it does not relate to a specific stressful experience. The participants evaluated how much they were bothered by the specific problem of the COVID-19 lockdown in the previous month. In each of the 17 items, the following expression was added: “a stressful experience from the COVID-19 lockdown” (e.g., Item 1: *Repeated, disturbing memories, thoughts, or images of a stressful experience from the COVID-19 lockdown*). Cronbach’s α for COVID-19-related PCL-S in this study was 0.96 in the total sample at T1 and T2.

#### 2.3.2. Perceived Stress

For the measurement of perceived stress, we used the Perceived Stress Scale (PSS-10) [32]. It measures whether participants appraised the situation in their life as stressful [33]. The scale consists of 10 items regarding the frequency of stressful events that occurred in the month preceding the study. Items are evaluated on a five-point scale (0 = never to 4 = very often). The result is a sum of points. The total score ranges from 0 to 40, with 0 to 13 points is considered as low perceived stress, 14 to 19 as medium, and 20 to 40 as high. Cronbach’s α for PSS-10 in this study was 0.83 at both T1 and T2.

#### 2.3.3. Fear of Vaccination 

The Fear of Vaccination (FoVac) [34] is based on FCV-19S [35]. The term “coronavirus-19” was changed to “vaccination for COVID-19” for each item. This methodology has shown to be successful in previous works, e.g., adaptation of the COVID-19 Perception Risk Scale (CRPS) from the SARS Risk Perception Scale (SRPS) [36] and the Spanish six-item Vaccination Fear Scale (VFS-6) [20]. FoVac consists of seven items and uses a five-item Likert-type scale (from 1 = strongly disagree to 5 = strongly agree), similar to FCV-19S. The total score ranges from 7 to 35. The higher the score, the greater the fear of vaccination for COVID-19. Cronbach’s α for fear of vaccination was 0.92 at T1 and 0.93 at T2 in the total sample in this study.

#### 2.3.4. Sociodemographic Data

Regarding sociodemographic data [34], the survey in this study included gender, age, place of residence, employment status, relationship status, and having children. 

### 2.4. Statistical Analyses

The initial analyses of the descriptive statistics demonstrated suitable psychometric properties for coronavirus-related PTSD, perceived stress, and fear of vaccination, at both timepoints (T1 and T2) during the pandemic. The basic assumptions for parametrical tests (normality of distribution, homogeneity of variance, linearity, independence) were met. Therefore, parametric analyses were conducted in the following steps of the study. A paired-samples Student’s *t*-test was used to test differences among all continuous variables (coronavirus-related PTSD, stress, and fear of vaccination) between the first and second measurement timepoints. The Cohen’s d coefficient was used to assess effect size (small when *d* = 0.20, medium when *d* = 0.50, large when *d* = 0.80). In addition, a repeated measures two-way ANOVA was performed to examine changes in these variables over time (differences between T1 and T2 for the same variable) across four groups representing the following countries: Germany, Israel, Poland, and Slovenia. Effect size was estimated using the η^2^*_p_* coefficient (small effect if η^2^*_p_* > 0.01, medium if η^2^*_p_* > 0.06, large effect if η^2^*_p_* > 0.14). The statistical analyses, as well as the creation of all figures, were carried out using JASP Team [37].

G*Power [38] was used to calculate the appropriate sample size. A minimum of 54 participants was required for matched pairs, two-tailed Student’s *t*-test, with Cohen’s *d* = 0.50 effect size at the alpha of 0.05 and 95% power. For repeated measures two-way mixed factor analysis of variance (ANOVA), the expected sample size was 158, assuming two groups and two measurements, effect size *f*^2^ = 0.25, repeated measures *r* = 0.50, *p* < 0.05, and 95% *CI*. 

## 3. Results

### 3.1. Changes in Mental Health during the Third Wave of the COVID-19 Pandemic in the Total Sample

**Figure 2:**
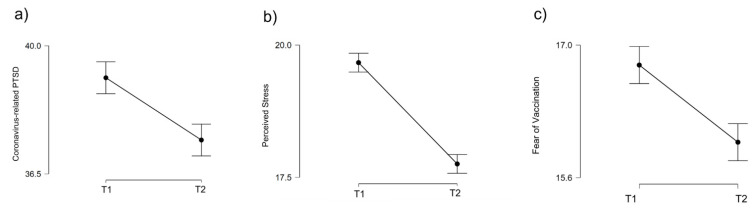
Changes in mental health indices: (**a**) coronavirus-related post-traumatic stress disorder (PTSD); (**b**) perceived stress; and (**c**) fear of vaccination among young adults at the first measurement timepoint (T1) in February 2021 and the second measurement (T2) in May–June 2021.

A paired-samples Student’s *t*-test was carried out to examine changes over time in mental health indices: coronavirus-related PTSD, perceived stress, and fear of vaccination. The results are shown in Table 1. Significant decreases in coronavirus-related PTSD, perceived stress, and fear of vaccination were found at T2, compared to T1. The effect size was small for all variables in this study (see Table 1 and Figure 2 for more details). 

### 3.2. Changes in Mental Health during the Third Wave of the COVID-19 Regarding Time, Country and Time–Country Interaction

When country was included in the analysis as a factor variable, a repeated measures ANOVA showed a significant main effect for Time (T) (differences between T1 and T2), Country (C) (differences between samples from Germany, Israel, Poland, and Slovenia), and interaction between change over time and countries in all variables, excluding interaction effects for coronavirus-related PTSD and perceived stress (Table 2). 

The effect sizes for T, C, and interaction between Time and Country (TxC) were small for most variables, except a medium effect size for C in coronavirus-related PTSD and fear of vaccination, as well as a medium effect size for T in perceived stress. TxC interaction was insignificant for coronavirus-related PTSD and perceived stress (see Table 2 for more details).

## 4. Discussion

Our study demonstrated a significant decrease in coronavirus-related PTSD, perceived stress, and fear of vaccination during a three-month period at the point of the third wave of the COVID-19 pandemic among a representative sample of 1723 young adults from Poland, Slovenia, Israel, and Germany. The study revealed the significance of country, timepoint, and interaction between country and time effects for mental health indices in four countries. 

### 4.1. Change in Fear of Vaccination

We revealed a small effect of time, a medium effect of country, and a small effect of the interaction between country and time for fear of vaccination. 

The lowest fear of vaccination was observed in Israel, while the highest was in Poland. Considering interaction, the highest fear of vaccination was at T1 in Poland, while the lowest was in Israel at T2. However, although significant, this was a small effect. We expected that the vaccination process in each country would play a substantial role. Even though the number of vaccinated people in Israel at our first measurement (T1) timepoint significantly exceeded the numbers recorded in other countries, a noticeable increase at the second measurement (T2) timepoint was observed in Poland, Slovenia, and Germany [25]. Our study showed that along with the increase in the number of vaccinated people, fear of vaccination substantially dropped over the three-month period. This was mainly observed in Israel, where the number of vaccinated people was the highest while the fear of vaccination was the lowest. In Poland, the highest rates of fear of vaccination along with the lower number of vaccinated people can be explained by additional external circumstances, such as the strong objections against vaccination due to ethical issues from Polish Catholic Church representatives [39]. On the other hand, the Israeli efforts to promote vaccination were highly successful due to a combination of facilitating factors, e.g., health system organization, IT and organizational capacities, and COVID-19 vaccination efforts [40].

### 4.2. Change in Perceived Stress and Coronavirus-Related PTSD

The decrease in stress and coronavirus-related PTSD was significant, although small, in this study. Our findings are in line with recent longitudinal research showing that more general mental indices, such as anxiety and depression, were relatively stable over time, while, fear of COVID-19 systematically fluctuated in line with mortality rate and social restrictions [41]. Our previous work on the difference in prevalence of anxiety and depression risk among young adults similarly revealed stability over time [21]. A repeated cross-sectional study among 1961 Polish students [42] showed a large decrease in perceived stress during the second and third waves of the COVID-19 pandemic compared to the first wave. However, there was no significant change between the second and the third wave. The present research shows that in Poland, among a representative sample of young adults, perceived stress was significantly lower at T2 compared to T1, even though the third wave of the COVID-19 pandemic started between the measurement timepoints (T1 and T2). Perceived stress decreased significantly during the present study in all countries. Despite the small effect, this change was particularly significant, since the levels of stress in all countries at T1 were high, while at T2, they were medium. 

The mean value for coronavirus-related PTSD also significantly decreased at T2 (37.45) compared to T1 (39.13), in this cross-national representative sample of young adults. The mean value for PTSD related to COVID-19 in a previous cross-national study among students (during the second wave of the COVID-19 pandemic) was 38.08 [11], similar to the values in the present study. These mean values are interpreted as moderate to moderately high severity of PTSD symptoms, and psychotherapeutic help is recommended [30]. Hence, even though we found a significant decrease in coronavirus-related PTSD, psychological intervention is still required. 

The lowest coronavirus-related PTSD levels were found in Israel, while the highest were found in Germany. The significantly lower level of coronavirus-related PTSD in Israel may be because of mental health policy, the economy, and political stability [43]. Due to the high volatility of the Israeli–Palestinian conflict, the Israeli mental health care system offers easy access to resilience centers that promote mental health and mental health interventions [44,45]. However, even though the level of coronavirus-related PTSD was lower in Israel, it still reached moderate to moderately high severity levels, similar to Poland, Slovenia, and Germany. The German sample of young adults showing the highest results in terms of coronavirus-related PTSD can be interpreted in light of Germany’s high prevalence of depression. There is a positive link between PTSD and depression that commonly co-occurs [46]. German young adults also present higher depression prevalence compared to young adults in Poland, Slovenia, and Israel [21]. Furthermore, these depressive symptoms are more frequent when compared to the European Union average, particularly among young adults [47]. Additionally, women manifest depression symptoms more frequently than men in Germany, due to cumulating psychosocial factors [47]. Our previous research among university students from six countries showed that female gender, previous diagnosis of depression along with exposure to COVID-19—death of loved ones, job loss, and decrease in economic status due to the pandemic—were risk factors for high risk of coronavirus-related PTSD [11]. Yet, during the pandemic, massive economic support was provided to citizens in Germany, so the likelihood of the deterioration of one’s financial situation and/or job loss was significantly reduced compared to other European countries [48]. Furthermore, German university students did not experience a negative impact on their financial situation between the first and the second wave of the COVID-19 pandemic [11]. Therefore, higher coronavirus-related PTSD among German young adults can be related to depression severity instead, rather than economic factors. 

### 4.3. Limitations

There are some limitations in the present study. PCL-S, based on DSM-4, was used in this study instead of PCL-5, based on DSM-5. However, PCL-S enables the measurement of PTSD with regard to a specific stressful experience: the COVID-19 pandemic. Furthermore, all variables were evaluated by means of self-assessment measurements, which can be a source of bias. Furthermore, the study was conducted under specific pandemic circumstances, which can undermine the reliability of this study. 

At the time of writing, the COVID-19 pandemic is not nearing its end; thus, there is a need to gain insight into young adults’ unique experience in order to raise awareness among public health professionals, educators, and policy makers of the high prevalence of psychological distress experienced by young adults. In line with this, Clotworthy et al. [49] highlighted important individual variations in perceptions of lockdown and, thus, the necessity to study both qualitative and quantitative data to understand individual reactions to the pandemic better. In contrast, our study only implemented a quantitative approach.

## 5. Conclusions

On the verge of the third wave of the COVID-19 pandemic, our longitudinal study revealed an adaptation to the volatile pandemic situation, which was observed in positive changes in mental health indices, such as perceived stress, coronavirus-related PTSD, and fear of vaccination. Even though these mental health indices differed between countries, perceived stress and coronavirus-related PTSD were above the minimal levels across all countries. The novelty of this study is how it reveals changes in mental health indices from cross-cultural and longitudinal perspectives among representative samples of young adults. This is the first study to show fear of vaccination from this perspective. Our results show that it is necessary to create tailored services (e.g., emergency mental health crisis support or remote interventions such as telemental and telebehavioral health services, which may be more suitable for young adults [6,50]) to address the psychological distress that young adults are facing. Coronavirus-related PTSD in Germany and perceived stress, as well as fear of vaccination in Poland, should be taken into consideration with particular care.

## Figures and Tables

**Table 1:** Differences in mental health indices between T1 and T2 among young adults (*N* = 1723).

	Time 1		Time 2				Cohen’s *d*
Variable	*M*	*SD*	*M*	*SD*	*t*(1722)	*p*	
Coronavirus-related PTSD	39.13	15.93	37.43	15.88	5.42	<0.001	0.13
Stress	19.67	6.59	17.75	5.83	14.96	<0.001	0.36
Fear of Vaccination	16.79	7.11	15.97	7.20	5.77	<0.001	0.14

*Note.* Time 1 = measurement in February 2021; Time 2 = measurement in May–June 2021.

**Table 2:** Mean, standard deviation, and repeated measures two-way ANOVA statistics for study variables in representative samples of young adults from Germany, Israel, Poland, and Slovenia.

			Country												
			Germany (*n* = 418)		Israel (*n* = 428)		Poland (*n* = 446)		Slovenia (*n* = 431)						
Variable		Range	*M*	*SD*	*M*	*SD*	*M*	*SD*	*M*	*SD*	Effect	*F*	*df*	*p*	η^2^*_p_*
Coronavirus-related PTSD		17–85									T	29.77	1, 1719	<0.001	0.02
	Time 1		43.40	15.90	34.30	15.60	40.3	15.50	38.70	15.50	C	36.63	3, 1719	<0.001	0.06
	Time 2		41.70	15.60	31.20	14.60	39.4	15.80	37.40	15.60	TxC	2.45	3, 1719	0.062	<0.01
Perceived stress		0–40									T	223.44	1, 1719	<0.001	0.12
	Time 1		19.10	5.83	19.10	7.16	20.80	5.95	19.70	7.18	C	8.91	3, 1719	<0.001	0.02
	Time 2		17.10	5.06	17.40	6.32	18.90	5.32	17.60	6.35	TxC	0.34	3, 1719	0.794	0.00
Fear of vaccination		7–35									T	32.86	1, 1719	<0.001	0.02
	Time 1		17.10	7.02	14.20	6.76	18.90	7.15	16.80	6.68	C	40.90	3, 1719	<0.001	0.07
	Time 2		16.60	7.35	13.02	6.47	17.40	7.41	16.83	6.70	TxC	5.72	3, 1719	<0.001	0.01

*Note*. *N* = 1723. ANOVA = analysis of variance; T = Time of measurement; C = Country; TxC = interaction effect between Time and Country.

## Data Availability

This study is a part of an international research project “Mental health of young adults during the COVID-19 pandemic in Poland, Germany, Slovenia and Israel: a longitudinal study” [34], registered at the Center for Open Science (OSF). The datasets used and analyzed during the current study are available from the corresponding author upon reasonable request.
